# Evolving Trends in Surgical Management of Breast Cancer: An Analysis of 30 Years of Practice Changing Papers

**DOI:** 10.3389/fonc.2021.622621

**Published:** 2021-08-04

**Authors:** Stephen Keelan, Michael Flanagan, Arnold D. K. Hill

**Affiliations:** ^1^The Department of Surgery, The Royal College of Surgeons in Ireland, Dublin, Ireland; ^2^The Department of Surgery, Beaumont Hospital, Dublin, Ireland

**Keywords:** breast cancer, breast cancer surgery, mastectomy, axilla, breast conserving therapy

## Abstract

The management of breast cancer has evolved into a multidisciplinary evidence-based surgical speciality, with emphasis on conservative surgery. A number of landmark trials have established lumpectomy followed by radiation as the standard of care for many patients. The aim of this study is to construct a narrative review of recent developments in the surgical management of breast cancer and how such developments have impacted surgical practice. A comprehensive literature search of Pubmed was conducted. The latest search was performed on October 31^st^, 2020. Search terms “breast cancer” were used in combinations with specific key words and Boolean operators relating to surgical management. The reference lists of retrieved articles were comprehensively screened for additional eligible publications. Articles were selected and reviewed based on relevance. We selected publications in the past 10 years but did not exclude commonly referenced and highly regarded previous publications. Review articles and book chapters were also cited to provide reference on details not discussed in the academic literature. This article reviews the current evidence in surgical management of early-stage breast cancer, discusses recent trends in surgical practice for therapeutic and prophylactic procedures and provides commentary on implications and factors associated with these trends.

## Introduction

Breast surgery is a complex multi-disciplinary surgical specialty. The breast surgeon must diagnose and treat breast cancer in symptomatic patients and coordinate the timing of surgery as dictated by systemic and radiation therapies. Treatment varies on a case-by-case basis from breast conserving surgery to mastectomy to specialized oncoplastic techniques and reconstructive procedures. Since the first Halsted radical mastectomy the range of surgical approaches has increased greatly. Following the introduction of the modified radical mastectomy it took almost 30 years for breast conserving surgery and adjuvant radiotherapy became an accepted standard of care (1).

Breast surgeons further challenged breast conserving surgery (BCS) in pursuit of improving cosmesis while maintaining oncological outcomes. This paradigm shift towards better cosmetic outcomes and quality of life led to the advent of oncoplastic surgery (2).

This paper will discuss the advances in the surgical management of breast cancer over the last 30 years while also providing an overview of emerging surgical options and the future they bring to the sphere of breast cancer management.

## From Mastectomy to Breast Conservation

Breast surgery has undergone significant changes over time. First, Halsted’s radical mastectomy gained widespread acceptance as the standard of care up until 1960's. While this procedure improved local control, the extensive dissection of skin, breast, pectoralis muscles and axillary contents caused significant morbidity (3). Furthermore, to improve its curative potential some surgeons also excised the internal mammary nodes. This became known as an extended radical mastectomy. However this did not improve patient survival (4, 5).

To reduce morbidity, Patey introduced the modified radical mastectomy (MRM) excising the breast, pectoralis major fascia, and level I and II axillary lymph nodes (6). At the same time McWhirter introduced the simple mastectomy which combined surgery with radiotherapy. Several randomised controlled trials investigated survival outcomes of these two methods compared to Halsted’s radical mastectomy. The National Surgical Adjuvant Breast and Bowel Project (NSABP) B-04 trial observed no significant improvement in survival for patients treated with Halsted radical mastectomy compared to less extensive surgery. NSABP B-04 also found the addition of local-regional radiation to total mastectomy had no significant advantage in overall survival (OS). Additionally, it found that in node negative disease, routine axillary lymph node dissection (ALND) is overly aggressive (7). As such, this trial heralded the move toward increasingly conservative surgical management of breast cancer along with introducing the first concept of multi-modality therapy.

The NSABP B-06 trial was the first trial to establish BCS as a feasible treatment option for early invasive breast cancer when used in conjunction with radiation (8). No significant difference in OS or disease-free survival (DFS) was found in patients receiving BCS with or without radiation compared to those receiving modified radical mastectomy. The rate of local regional recurrence (LRR) was significantly higher in those who underwent lumpectomy without radiation (8).

**Figure 1:**
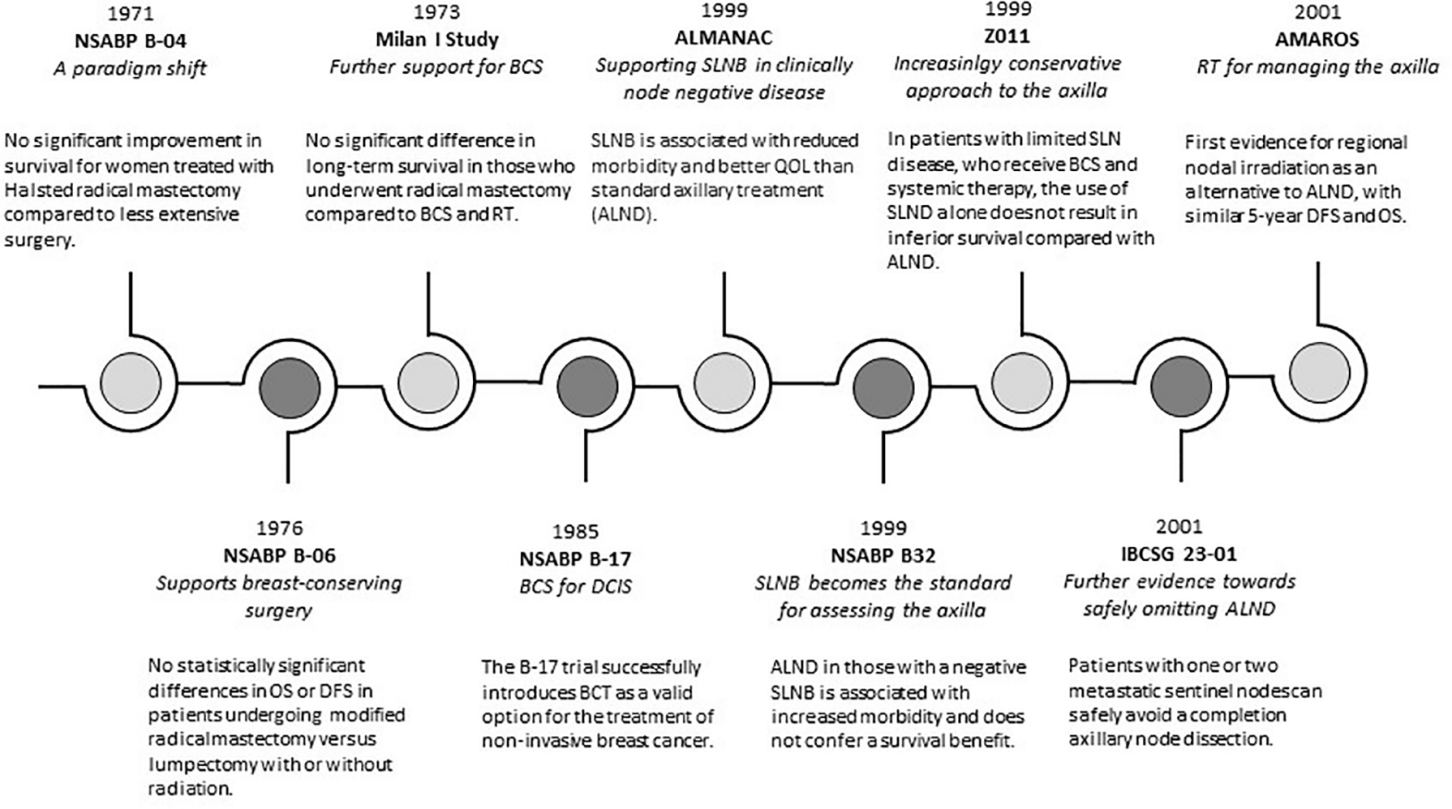
A timeline of evolving trends in surgical management of breast cancer. OS, overall survival; DFS, disease free survival; BCS, beast conserving surgery; RT, radiotherapy; QOL, quality of life; SLNB, sentinel lymph node biopsy; ALND, axillary lymph node dissection.

The Milan Cancer Institute (Milan I Study) further established BCS as the standard of care for early breast cancer (≤2cm in diameter). Despite higher local recurrence in the BCS group, there was no significant difference in long-term survival in those who underwent radical mastectomy compared to BCS and radiotherapy (1). **Table 1** outlines the landmark randomised controlled trials (RCT) in the surgical management of non-invasive and invasive breast cancer. **Figure 1** is a timeline of landmark trials in the surgical management of breast cancer.

**Table 1:** Landmark RCT’s in the surgical management of the axilla.

Trial Name	Study years	No. Participants	Population Characteristics	Mean follow-up (months)	Intervention	Primary outcome
**Landmark RCT’s in the surgical management of the axilla**						
ALMANAC (58)	1999–2003	1031	Any tumor size and clinically node-negative breast cancer	12	ALND vs SLNB alone (if negative) or SLNB and ALND or axillary RT (if positive)	Arm and shoulder morbidity and QOL: SLNB was associated with reduced arm morbidity and better QOL.
NSABP B32 (46)	1999–2004	5611	<4 cm invasive breast cancer and clinically node-negative breast cancer	96	SLNB + ALND *vs* SLNB alone (if negative)	OS: No significant difference DFS: No significant difference Axillary recurrence: No significant difference
**Landmark RCT’s comparing ALND with no further treatment for patients with positive SLNB**						
Z0011 (51)	1999–2004	856	T1-2 breast cancer, and 1-2 metastatic nodes by SLNB. All underwent lumpectomy and whole-breast irradiation	76	ALND vs No further axillary treatment	OS: No significant difference DFS: No significant difference
IBCSG 23-01 (48)	2001–2010	931	<5 cm invasive breast cancer and 1 or more micrometastatic sentinel nodes	60	ALND vs No further axillary treatment	OS: No significant difference DFS: No significant difference
**Landmark RCT’s comparing ALND with axillary radiotherapy for patients with positive SLNB**						
AMAROS (55)	2001–2010	4805	T1-2 primary breast cancer and no palpable lymphadenopathy	73	ALND vs Axillary radiation	OS: No significant difference DFS: No significant difference Axillary recurrence: 0.43% ALND vs 1.19% axillary radiation

SLNB, sentinel lymph node biopsy; ALND, axillary lymph node dissection; OS, overall survival; DFS, disease-free survival; QOL, quality of life.

BCS focuses on three primary aims; obtain tumour free margins, achieve a good cosmetic outcome, and at least equivalent survival to traditional mastectomy. As such the following contraindications must be considered before proceeding with BCS:

*-Multicentric disease* - Two or more primary tumours in different quadrants of the breast such that they cannot be removed with a single excision
*-*Presence of *diffuse malignant-appearing calcifications* on imaging (mammogram or magnetic resonance imaging [MRI])
*-Previous history of chest radiotherapy* - which, when combined with the proposed treatment, would result in an excessively high total radiation dose to the chest wall
*-Pregnancy*
*-Persistently positive margins* despite attempts at re-excision

Furthermore, a consideration, but not an absolute contraindication to BCS is a large tumour in a relatively small breast. Neoadjuvant chemotherapy (NACT) is increasingly used in these patients for the purpose of downstaging the tumour and thus, making the patient eligible for BCS (12–14). Notably when compared to adjuvant chemotherapy, those receiving NACT do not benefit in terms of survival and local recurrence (12, 13, 15).

Local recurrence is a risk factor for distant metastasis (16). The local recurrence rate after BCS (2% at 10 years) is no longer considered higher than that after mastectomy (17, 18). Risk factors for local recurrence include young age, positive surgical margins, node positivity, estrogen receptor negativity, and absence of radiation therapy (19). Surgical margins are a controllable risk factor. Current recommendations for the adequacy of margins are based off a large meta analyses in 2014, which included 1506 ipsilateral breast tumour recurrences (IBTRs) (20). At a median follow-up of 79 months, the median prevalence of IBTR was 5.3%. A positive margin, defined as “ink on tumour”, was associated with more than a two-fold increase in IBTR. Routine re-excision is not necessary for close positive margins (e.g. <1 mm), however clinical and pathological features should guide decisions to perform a second operation (21, 22). Positive margins are associated with a two-fold increase in LRR (20) and necessitate reoperation. Rates of reoperation vary from less than 10% to more than 50% (23–25).

## IncreasING Mastectomy Rates

It was expected that rates of mastectomy would decrease with the availability of screening mammography. However, the effect of screening on surgical treatment has yielded conflicting results (26, 27). Increasing rates of prophylactic mastectomies may partially account for unchanged mastectomy rates, offsetting the benefits of advances in BCS (28). Improvements in reconstruction options have brought about an unanticipated increase in contralateral prophylactic mastectomy rates. A once disfiguring procedure, patients and surgeons are now more aware of symmetry and cosmesis post-surgery. Low satisfaction scores among patients undergoing unilateral mastectomy with implant-based reconstruction suggests cosmetic factors may be a driver of increasing contralateral prophylactic mastectomy rates (29, 30).

Furthermore, some patients with early-stage breast cancer who are suitable for BCS, choose to undergo mastectomy instead. While the reasons for this are unclear, they may in part be attributed to a fear of recurrence, thus triggering a move towards more “aggressive” management approaches. However, it is important to note in young patients with early-stage breast cancer, BCS with adjuvant radiotherapy has comparable OS to mastectomy alone (31). This has been seen in a number of studies which have demonstrated improved OS and DFS in BCS compared to mastectomy (32–38). BCS may in fact have superior LRR compared to mastectomy due to a number of factors (39), including developments in radiation treatment planning which have resulted in increased coverage of residual breast tissue compared to techniques in original trials. Improvements in imaging modalities have resulted in more accurate selection of patients for BCS i.e. those without multicentric disease. Finally, with newer less invasive mastectomy techniques gaining popularity, it is conceivable that techniques such as nipple/skin sparing mastectomy are being adopted in patients that have less favourable tumour characteristics than those in the studies in which these approaches were initially assessed (40).

## Management of the Axilla

Management of the axilla has evolved in the last decade. Axillary nodal metastasis is a significant prognostic factor in breast cancer, influencing surgical and adjuvant treatment (41, 42). While the surgical approach to the axilla has become increasingly conservative, the optimal management of the axilla continues to be a controversial topic.

Traditionally all patients proceeded to ALND irrespective of nodal status (43). ALND is associated with significant morbidity including lymphedema, impaired shoulder movement and arm sensation, resulting in a considerable impact on quality of life (44, 45). The NSABP B32 trial randomized 5611 patients with clinically node-negative disease and a negative SLNB into two groups, ALND *versus* no further treatment. It found no significant difference in OS, DFS, or LRR between both groups. This demonstrated that ALND in those with a negative SLNB does not confer any survival benefit (46). SLNB was ultimately established as optimum standard for surgically assessing the axilla.

The extent of metastatic disease within the SLN is of prognostic importance. Nodal involvement is classified as macro-metastatic (>2mm), micro-metastatic (<2mm) or as isolated tumour cells (ITC). A systematic review found that the presence of micro-metastases is associated with decreased OS (47). The IBCSG 23-01 (48) and the AATRM 048 (49) trials, in which the majority of patients received adjuvant systemic therapy, demonstrated that ALND does not confer survival benefits in those with micro-metastatic nodal disease. As a result, many surgeons now omit ALND in patients with ITC or micro-metastatic disease on SLNB.

In cases of macro-metastatic disease, ALND has remained the standard of care (50). However, the ACOSOG Z0011 (51) questioned whether this represented overtreatment. In this phase 3 non-inferiority trial, 856 patients with T1 to T2 tumours with less than 2 positive SLNs were randomized to ALND *versus* no ALND, after breast conserving surgery (BCS), SLNB, and adjuvant whole-breast irradiation (WBI). The 5-year OS was higher in the SLNB group compared to those receiving ALND (92.5% *versus* 91.9% respectively). The 5-year DFS was also higher in the SLNB group (83.9%) compared to the ALND group (82.2%). While not significant, the 10-year LRR was 5.3% in the SLNB group, *versus* 6.2% in the ALND group. These results have been practice-changing for many surgeons. However, the Z0011 results have also added to the controversy surrounding optimal management of the axilla (52–54). This comes from the fact that Z0011 inclusion criteria were set at patients with tumours up to 5cm in size who underwent BCS and received WBI postoperatively. Furthermore, this study also failed to enrol the planned number of patients and thus did not have sufficiently high power to detect small differences between the groups.

As the approach to the axilla continues to evolve, the use of an oncologically safe alternative to ALND has been investigated. The AMAROS (55) trial included 4806 patients with T1 to T2, clinically node-negative invasive breast cancer and a positive SLNB. Patients were randomized to receive ALND or regional nodal irradiation (RNI). All underwent BCS followed by WBI, or mastectomy with or without chest wall irradiation. This trial provided evidence for regional nodal irradiation (RNI) as an alternative to ALND, with similar 5-year DFS and OS. The Edinburgh trials (56) randomized patients with N1 disease into ALND *versus* SLNB with RNI. This trial reported a significant difference in LRR, which was not seen in the AMAROS trial, concluding that there was no significant difference in OS between ALND and RNI. Now, several countries offer axillary radiotherapy as an alternative to ALND. The POSNOC trial aims to add to the evidence for radiotherapy in axillary management in patients with macro-metastatic nodal disease undergoing BCS and systemic therapy (57). **Table 2** outlines the landmark RCTs in the surgical management of the axilla.

**Table 2:** Landmark RCT’s in the surgical management of the axilla.

Trial Name	Study years	No. Participants	Population Characteristics	Mean follow-up (months)	Intervention	Primary outcome
**Landmark RCT’s in the surgical management of the axilla**						
ALMANAC (58)	1999–2003	1031	Any tumor size and clinically node-negative breast cancer	12	ALND vs SLNB alone (if negative) or SLNB and ALND or axillary RT (if positive)	Arm and shoulder morbidity and QOL: SLNB was associated with reduced arm morbidity and better QOL.
NSABP B32 (46)	1999–2004	5611	<4 cm invasive breast cancer and clinically node-negative breast cancer	96	SLNB + ALND *vs* SLNB alone (if negative)	OS: No significant difference DFS: No significant difference Axillary recurrence: No significant difference
**Landmark RCT’s comparing ALND with no further treatment for patients with positive SLNB**						
Z0011 (51)	1999–2004	856	T1-2 breast cancer, and 1-2 metastatic nodes by SLNB. All underwent lumpectomy and whole-breast irradiation	76	ALND vs No further axillary treatment	OS: No significant difference DFS: No significant difference
IBCSG 23-01 (48)	2001–2010	931	<5 cm invasive breast cancer and 1 or more micrometastatic sentinel nodes	60	ALND vs No further axillary treatment	OS: No significant difference DFS: No significant difference
**Landmark RCT’s comparing ALND with axillary radiotherapy for patients with positive SLNB**						
AMAROS (55)	2001–2010	4805	T1-2 primary breast cancer and no palpable lymphadenopathy	73	ALND vs Axillary radiation	OS: No significant difference DFS: No significant difference Axillary recurrence: 0.43% ALND vs 1.19% axillary radiation

SLNB, sentinel lymph node biopsy; ALND, axillary lymph node dissection; OS, overall survival; DFS, disease-free survival; QOL, quality of life.

Despite this shift towards a conservative approach, some studies have raised the possibility that failure to remove nodal disease could be harmful. Park et al. (59) suggest that the rate of axillary recurrence among patients with a positive SLNB who did not undergo ALND was 2.0% at 30 months *versus* 0.4% in those receiving ALND. Additionally, a retrospective review of 257,157 patients diagnosed with breast cancer in the Surveillance, Epidemiology, and End Results (SEER) database revealed decreased survival in patients with stage IIA or higher disease with increased number of positive nodes and increased ratio of positive to total nodes removed (60).

Considering the conflicting data, many ongoing trials aim to clarify the aforementioned studies and strengthen the rationale for omitting extensive axillary surgery. The SENOMAC trial (61) is comparing ALND *versus* no ALND after surgery with the primary endpoint being DFS at 5 years. Coming almost full circle, some clinicians are examining the utility of SLNB itself. For example there is a growing interest in omitting SLNB in early breast cancer patients with a clinically and radiologically negative axilla (62, 63). However, other studies caution that despite a radiologically negative axilla there is a risk of high nodal burden axillary metastasis, particularly in T2 tumours. As such these patients should continue to undergo SLNB (64). Surgeons await the results from two RCTs, both the SOUND trial (Sentinel Node Vs Observation after Axillary Ultrasound) (NCT02167490) and the Intergroup-Sentinel-Mamma (INSEMA) trial (NCT02466737) which examine the role of AUS and SLNB in early breast cancer. It is possible that these trials will help negate surgical biopsy requirements in select patient groups, therefore advancing conservative axillary management further (65, 66). Whether we can omit the ALND from the management of patients with breast cancer altogether remains to be seen. However, the trajectory to date has seen the management of the axilla evolve from a low threshold for performing ALND to an increasingly conservative one, consequently improving morbidity and patient outcomes.

## Oncoplastic Surgery and Reconstruction

The primary aim of breast cancer surgery is complete tumour excision. However, improved cosmetic outcomes achieved with breast reconstruction continues to positively affect patient quality of life (67). This has given rise to the concept of oncoplastic breast surgery, which aims to provide an acceptable breast appearance while maintaining oncological effectiveness.

A variety of oncoplastic procedures have been described, and location of cancer within the breast is a major determinant of procedure choice (68–70). A 2014 meta-analysis found that patients treated with oncoplastic resections had a lower rate of positive margins (12% versus 21%) and a lower rate of re-excisions (4% versus 15%). Although patients undergoing oncoplastic surgery had a higher rate of completion mastectomies compared with those who underwent BCS (7% vs 4%), oncoplastic resections produced a higher satisfaction with breast appearance then standar BCS (90% vs 83%) (71–73). Furthermore, patients who underwent oncoplastic resections developed fewer complications (16% vs 26%) and decreased rates of local recurrence (4% vs 7%) at 3-5 year follow up, demonstrating that the long-term outcomes of oncoplastic surgery are comparable, if not better than standard BCS (71).

One of the first oncoplastic procedures that came into practice was the skin-sparing mastectomy (SSM), in which the breast parenchyma is excised, and most of the breast skin envelope is maintained (74). SSM has become a popular choice of procedure for patients with DCIS, early stage breast cancer as well as high-risk patients opting for prophylactic mastectomy due to its excellent cosmetic outcomes and acceptable oncological safety profile when compared to conventional mastectomy without reconstruction. Another commonly performed procedure is the nipple sparing mastectomy (NSM), used for high-risk women undergoing prophylactic surgery and also in select patients undergoing therapeutic mastectomy (75). This procedure preserves the nipple-areolar complex but removes major ducts from within the nipple lumen (76). A meta-analysis in 2018 demonstrated comparable 5 year DFS and LRR between NSM and SSM (77). Equally in a 2015 meta-analysis the OS, DFS, and LR rates of NSM were comparable to modified radical mastectomy and SSM (78).

Breast reconstruction can be performed using several techniques including an expander/implant and/or autologous tissues. Opinion within the surgical community regarding immediate breast reconstruction has evolved over time (79, 80). When planning the optimal reconstructive option, surgeons must consider patient-specific factors such as likelihood of postoperative radiation, prior breast radiation as well as patient preference. Typically, delayed reconstruction is indicated when there is impaired perfusion of the skin flaps post-mastectomy or when post-mastectomy radiotherapy will be needed (81). However, the absolute contraindication of immediate autologous reconstruction due to the challenges posed by post-mastectomy radiotherapy is increasingly being questioned. While radiotherapy after immediate autologous reconstruction had been thought to have a detrimental impact on flap outcome, several systematic reviews have shown no significant differences in measurable postoperative complications when comparing irradiated versus non-irradiated reconstructions. As such, immediate DIEP flap reconstruction in patients who need post-mastectomy radiation is an acceptable treatment option (82, 83). In the setting of inflammatory breast cancer where the presence of dermal lymphatic invasion often requires skin excision, a delayed reconstruction is more appropriate. However, often in cases of inflammatory breast cancer a decision is made not to proceed with reconstruction altogether.

## Risk Reducing Surgery

A growing list of breast cancer susceptibility genes accompanies the ever-increasing amount of published clinical data. High-penetrance breast cancer susceptibility gene mutations associated with inherited breast cancer syndromes, such as BRCA1, BRCA2, PTEN (Cowden’s syndrome), TP53 (Li Fraumeni syndrome), STK11 (Peutz-Jeghers syndrome), CDH1 (hereditary invasive lobular breast-diffuse gastric cancer) and those with an associated family history account for approximately 10% of breast cancers (84). BRCA1/2 mutations occur in 3-4% of all patients with breast cancer and in 10% of those with triple negative breast cancer (85, 86). Moderate penetrance breast cancer susceptibility gene mutations such as PALB2, CHEK2, ATM occur in 4-6% of breast cancer patients (85). Generally, it is advised that high-risk patients undergo more frequent screening, use of imaging modalities and consider prophylactic risk reducing surgery. Recently published guidelines offer recommendations on the management of breast cancer in patients with germline mutations in BRCA1/2, PALB2, CHEK2 and ATM (87).

Bilateral prophylactic mastectomy reduces the risk of breast cancer by 95% in patients with BRCA 1&2 mutations, and by 90% in those with a strong family history of breast cancer (88). Prophylactic mastectomy may be performed using many of the techniques described. Contralateral prophylactic mastectomy is considered for patients with a high lifetime risk for developing contralateral breast cancer, such as BRCA mutations, strong family history, or young patients with aggressive disease (87). Bilateral prophylactic salpingo-oopherectomy can reduce the risk of ovarian cancer by approximately 80% and the risk of all-cause mortality by 68% (89). Decisions regarding prophylactic mastectomy must be individualized for every patient. Benefits of the reduced anxiety relating to developing breast cancer must be balanced against risks of surgery, complications from reconstructive surgery as well as any potential adverse feelings relating to body image.

As family history breast clinics are further incorporated into routine clinical practice worldwide and as next-generation sequencing continues to become more accessible, it is expected that there will be an increase in the number of BRCA1/2 mutations diagnosed each year and at an earlier age. Thus, forward planning by policy makers for the provision of all aspects of patient management, including genetic counselling, surgery, radiotherapy, and oncological therapy, are required.

## Novel Therapeutics

### Interventional Radiology (IR)

The use of IR-guided cryoablation as a minimally invasive technique to treat primary breast tumours is being explored (90). Through repetitive freezing/thawing cycles or rapidly decompressing argon gas, cryoablation results in cell injury and coagulative necrosis (91). Some studies have demonstrated feasibility of cryoablation for early breast cancer treatment (92, 93). Ongoing trials are investigating complete response rate and local recurrence without subsequent surgery (FROST trial – NCT01992250; Ice3 trial – NCT02200715). This emerging modality may be most useful in those with significant co-morbidities who are less suitable for surgical resection. Other image-guided ablation techniques include radiofrequency ablation, microwave ablation, high-intensity focused ultrasound, laser ablation and irreversible electroporation (94).

### Neoadjuvant Chemotherapy and Non Operative Strategies

Neoadjuvant treatments are increasingly being used in high-risk breast cancers such as triple negative and Her2 positive breast cancer. Neoadjuvant therapies are offered in patients at high risk of recurrence, in locally advanced disease, and to downstage the tumour to allow for BCS. Achieving a pathological complete response (pCR) is associated with improved event free survival and overall survival, particularly in triple negative and Her2 positive breast cancer (95, 96).

Patients who achieve a partial or complete response pose a clinical dilemma in applying established surgery and radiotherapy treatment protocols. Patients who demonstrate a good clinical response to neoadjuvant treatment may benefit from de-escalation strategies in the adjuvant setting based on the degree of neoadjuvant response. Optimal methods to accurately detect a complete pathological response and the oncological safety in de-escalation strategies are currently the focus of a number of trials.

One such de-escalation strategy is to provide BCS for patients previously deemed unresectable or unsuitable for BCS. In an era of targeted therapy, increased rates of pCR in the breast have been observed. However advances in response to systemic therapy have not been matched with increased rates of BCS. It would be expected that those who achieve a complete response would be more likely to undergo BCS. However meta-analysis of RCT assessing eligibility for BCS following neoadjuvant chemotherapy found no association between rates of BCS and pCR (97). The inability to accurately detect viable tumour following neoadjuvant chemotherapy may contribute to the decision of the surgeon to perform a less radical procedure.

De-escalation of axillary management after neoadjuvant chemotherapy has also been explored following high rates of nodal pCR in patients who have histologically confirmed nodal disease (98, 99). Due to the increased likelihood of false negative sentinel node biopsy following neoadjuvant chemotherapy, de-escalation of axillary clearance to sentinel lymph node biopsy alone following neoadjuvant chemotherapy in patients who were previously clinically node positive should only be considered if 3 or more negative nodes have been retrieved.

Whether surgery can be omitted in patients receiving neoadjuvant treatment who obtain a pCR, is under investigation. A trial (NCT02945579) is evaluating patients with HER2 positive or triple negative breast cancer who forgo surgery after systemic neoadjuvant therapy.

There is currently no evidence to suggest that avoidance of surgery in patients who have a pCR is oncologically safe. Analysis of the NSABP B-18 and B-27 trials observed LRR of 6-9% in patients who had a pCR following neoadjuvant chemotherapy and BCS or mastectomy (100).

Until such a time as the accuracy of imaging and core needle biopsies can reliably determine pCR surgery with histological assessment of the resected specimen is likely to remain a corner stone of effective treatment, accurate assessment of pCR, and reduction of local regional recurrence.

## Future Perspective on Breast Cancer Surgery

Surgical innovation continues to drive advances in the management of breast cancer. Artificial intelligence (AI) technology and machine learning algorithms applied to diagnostic imaging and analysis of large clinical and genomic datasets in predicting response to treatment have been shown to improve patient outcomes (101–104). Once healthcare practitioners have overcome the fear of the unknown and data scientists and AI experts become more incorporated into healthcare, the future of surgical breast cancer management may change rapidly. Capabilities for storing vast amounts of data for imaging analysis can be applied to a multitude of areas from digital pathology to surgical planning. Digitization of breast cancer pathology with whole slide imaging has enabled the use of artificial intelligence machine learning algorithms to be applied to digital pathology. These advances in computer aided diagnostics have the potential to replace some of the expensive multi-gene assays (105, 106). Machine learning for image analysis will act as an adjunct to enhance human reporting, increase accuracy, and improve outcomes by predicting the likelihood of recurrent disease and dictating the optimum surgical intervention. AI have also been used to aid surgical planning using MRI based 3D reconstructions of the tumour within the breast (107).

Technological advancements in the surgical management of non-palpable breast lesions such as wire-free radar technology to provide real-time surgical guidance during breast surgery have demonstrated efficacy and are oncologically safe (108, 109). The emergence of imaging and probe-based devices to detect differences between normal and cancerous tissue have the potential to improve margins, reduce re-operation rates and avoid current labour-intensive intraoperative margin assessment techniques such as frozen section and specimen radiology. The intelligent knife (iKnife) utilizes rapid evaporative ionisation mass spectrometry of aerosol generated by electrocautery of tissue. This technique provides a rapid and effective method for identification and characterization of neoplastic tissue, guides resection *in vivo* and improves the quality of the surgical resection (110, 111). A future surgical model may include SLNB and axillary dissection with real time diagnosis for presence of axillary disease.

## Conclusion

Advances in the surgical management of breast cancer have favoured an increasingly conservative approach. This article reviews the current evidence in surgical management of early-stage breast cancer, discusses recent trends in surgical practice for therapeutic and prophylactic procedures and provides commentary on implications associated with these trends.

## Author Contributions

Literature research – SK, MF. Manuscript Preparation – SK, MF. Manuscript Review – SK, MF, AH. All authors contributed to the article and approved the submitted version.

## Conflict of Interest

The authors declare that the research was conducted in the absence of any commercial or financial relationships that could be construed as a potential conflict of interest.

## Publisher’s Note

All claims expressed in this article are solely those of the authors and do not necessarily represent those of their affiliated organizations, or those of the publisher, the editors and the reviewers. Any product that may be evaluated in this article, or claim that may be made by its manufacturer, is not guaranteed or endorsed by the publisher.
